# A new potential mechanism of action of tianeptine – the effect on microglial cell activation

**DOI:** 10.1186/2193-1801-4-S1-P43

**Published:** 2015-06-12

**Authors:** Joanna Slusarczyk, Ewa Trojan, Anna Piotrowska, Katarzyna Glombik, Joanna Mika, Agnieszka Basta-Kaim

**Affiliations:** Institute of Pharmacology, Polish Academy of Sciences, Poland

**Keywords:** tianeptine, microglia, inflammation

Tianeptine is an atypical antidepressant drug with proven efficacy, but still not fully understood mechanism of action. Recently it has been suggested that tianeptine may modulate inflammatory processes, however there is a lack of data on its influence on microglia - the main source of pro-inflammatory cytokines in the brain. Therefore this project aimed to investigate whether tianeptine can influence activation of microglial cells. We conducted our study in two experimental models: in vivo – in the hippocampus and frontal cortex of adult rats and in vitro in microglial cultures. Pregnant rats were subjected daily to 3 stress sessions from 14th day of pregnancy until delivery. Control pregnant females were left undisturbed in their homecages. Microglial cells were pre-treated for 30min with different concentrations of tianeptine and stimulated with LPS (100ng/ml). Next, expression of microglial activation markers and pro-inflammatory cytokines were evaluated. In the second part of experiments at 3 months of age, after behavioral verification, control and prenatally stressed rats were injected with tianeptine (10mg/kg i.p.) for 14 days. Next, biochemical studies were carried out on hippocampus and frontal cortex. We observed that in microglial pre-treatment with tianeptine (1-10uM) reduced the expression of microglial activation markers (CD40 and MHCII) and production of pro-inflammatory cytokines. Moreover, in adult animals subjected to prenatal stress (an animal model of depression) chronic tianeptine treatment inhibited microglial activation (decreased CD40 and CD68 expression) in both examined structures. In conclusion, our results show that tianeptine exerts anti-inflammatory properties suppressing microglial activation in both in vitro and in vivo experimental models.

